# Local adjustment of exodermis to nutrient deficiency in crop species

**DOI:** 10.3389/fpls.2026.1817677

**Published:** 2026-07-20

**Authors:** Ondřej Gargoš, Zuzana Bauriedlová, Renáta Klvaňová, Adam Homola, Radomíra Vanková, Ivana Raimanová, Petra Mašková, Marek Šustr, Aleš Soukup, Edita Tylová

**Affiliations:** 1 Department of Experimental Plant Biology, Faculty of Science, Charles University, Prague, Czechia; 2 Institute of Experimental Botany, Czech Academy of Sciences, Prague, Czechia; 3 Division of Crop Management Systems, Czech Agrifood Research Center, Prague, Czechia

**Keywords:** crops, exodermis, local adjustment, nutrient deficiency, root plasticity, split-root system

## Abstract

**Introduction:**

Translating insights from *Arabidopsis* to crops to support the breeding programs remains a major challenge in plant science, particularly for root traits controlling nutrient transport. Here, we investigated how nutrient deficiency affects the differentiation of exodermis, a protective apoplastic barrier of the outer root cortex, in multiple crops.

**Methods:**

Panel of exodermis-forming monocot and dicot species was examined, including the transformable barley cultivar *Hordeum* vulgare cv. Golden Promise and the model monocot *Brachypodium distachyon*. Plants were exposed to nutrient deficiencies under homogeneous and split-root hydroponic conditions.

**Results:**

Among the tested nutrients, nitrogen deficiency emerged as the most potent stimulus of exodermal differentiation, while phosphorus deficiency elicited similar but weaker responses. In split-root systems, most species showed localized enhancement of exodermal differentiation in roots directly exposed to N deficiency, including lateral roots. In *Zea mays*, localized N deprivation was associated with locally altered root apoplastic permeability, minor retranslocation of nutrients toward deficient roots, and hormonal changes (especially of abscisic acid. Exogenous ABA promoted exodermal differentiation in *Z. mays*.

**Discussion:**

Despite interspecific differences in exodermal differentiation, the selected species exhibited a largely conserved exodermal response to N deficiency, indicating the existence of a conserved mechanism regulating root transport properties under nutrient-limiting conditions.

## Introduction

The survival of terrestrial plants depends on their ability to acquire water and nutrients from heterogeneous soils, where resources are often scarce and unevenly distributed. Roots exhibit pronounced developmental and physiological plasticity to meet this challenge. They can follow moisture gradients (Eapen et al., 2005), proliferate into nutrient-rich patches (Walch-Liu et al., 2006), chemically modify the rhizosphere (Kuzyakov and Razavi, 2019), establish symbioses with microorganisms (Azaizeh et al., 1995), enhance uptake efficiency through membrane transporters with distinct kinetic properties (Gordon-Weeks et al., 2003; Misson et al., 2004), and adjust root transport characteristics via modulation of apoplastic barriers (Barberon et al., 2016; Armand et al., 2019; Namyslov et al., 2020).

Root apoplastic barriers, represented by endodermis and exodermis, are key structural determinants of root uptake selectivity (Enstone et al., 2003; Geldner, 2013). They restrict uncontrolled apoplastic movement of water and solutes and thereby contribute to the regulation of radial transport across the root. The endodermis is an obligatory inner cortical barrier (Enstone et al., 2003). The exodermis differentiates in the outer cortex and is widespread among angiosperms (Perumalla et al., 1990). It is structurally analogous to the endodermis, but its lignification and suberization are governed by regulatory programs that only partly overlap with those controlling endodermal differentiation (Kajala et al., 2021; Wang et al., 2022; Manzano et al., 2024). Because of its peripheral position at the soil–root interface, the exodermis protects the cortex from uncontrolled entry of solutes, toxic compounds, or pathogens, and contributes to nutrient uptake regulation (Meyer et al., 2009; Redjala et al., 2011; Andersen et al., 2015; Namyslov et al., 2020). The exodermis is more developmentally plastic than endodermis (Stasovski and Peterson, 1993; Enstone et al., 2003), and is important for plant stress tolerance (Ederli et al., 2004; Meyer et al., 2011; Cheng et al., 2020). Despite its functional significance, it remains comparatively understudied in breeding strategies targeting root traits (Liu and Kreszies, 2023).

The barrier function of the endodermis and exodermis is established through the formation of Casparian bands (CB) and suberin lamellae (SL). CB are lignified areas of radial and transversal cell walls that restrict apoplastic continuity, whereas SL deposit during the second phase of differentiation and form hydrophobic layers that reduce direct water and solute access from apoplast to plasmalemma (Enstone et al., 2003; Geldner, 2013). In nutrient acquisition, CB-bearing endodermal or exodermal layer function similarly to polarized epithelium in animals, highly active in selective nutrient uptake and radial transport (Barberon and Geldner, 2014). Deposition of SL then blocks the accessibility of nutrient transporters at the plasma membrane to nutrients moving through apoplast and restricts the uptake to symplast to more peripheral cell layers (Enstone et al., 2003).

CB and SL differentiate progressively along the root axis, gradually transforming root regions from absorptive to protective. Therefore, the position along the root axis, where these modifications are established behind root apex, is an important factor determining overall root system transport properties. The position is shifted by abiotic stress factors (Zimmermann and Steudle, 1998), e.g. low water availability (Zimmermann and Steudle, 1998; Meyer et al., 2011; Suresh et al., 2024), salinity (Walker et al., 1984; Cheng et al., 2020), toxic substances in soil (Lux et al., 2011; Huang et al., 2019), and nutrient availability (Barberon et al., 2016; Armand et al., 2019; Namyslov et al., 2020), which contributes to plant stress tolerance (Meyer et al., 2011).

In addition to other factors, the differentiation of barriers is regulated by phytohormones. Abscisic acid (ABA) stimulates endodermal suberization in *Arabidopsis thaliana* (Barberon et al., 2016; Wang et al., 2020; Shukla et al., 2021), upregulates suberin biosynthesis genes (Xu et al., 2022), regulates exodermal suberization in *Oryza sativa* under hypoxia (Shiono et al., 2021), and promotes exodermal differentiation in *Hordeum vulgare* (Shiono and Matsuura, 2024) and *Solanum lycopersicum* (Cantó-Pastor et al., 2024). In contrast, ethylene application delays endodermal suberization in *A. thaliana* and *Sedum alfredii* (Barberon et al., 2016; Liu et al., 2021). Phytohormones are also heavily involved in plant responses to nutrient stress (Peuke et al., 2002; Schraut et al., 2005) and in the regulation of root system architecture at both systemic (Jia et al., 2022) and local scales (Signora et al., 2001; Krouk et al., 2010).

Deficiencies of essential nutrients have frequently been shown to accelerate barrier differentiation (shifting it closer to the root apex), for example under low nitrogen (N) and phosphorus (P) in *Zea mays* and *H. vulgare* (Armand et al., 2019; Namyslov et al., 2020), low magnesium (Mg) in *Z. mays* (Pozuelo et al., 1984), and low potassium (K) or sulfur (S) in *A. thaliana* (Barberon et al., 2016). In contrast, delayed barrier differentiation has also been reported, such as under low N in *Ricinus communis* (Schreiber et al., 2005), low K in *Z. mays* (Namyslov et al., 2020), and iron (Fe), zinc (Zn), or manganese (Mn) deficiency in *A. thaliana* (Barberon et al., 2016).

In addition, split-root experiments with *Z. mays* demonstrated that individual roots within a single root system could locally adjust endodermal and exodermal development in response to nutrient availability in the surrounding medium (Namyslov et al., 2020). Such localized regulation is likely critical for fine-tuning whole-root-system transport properties in heterogeneous soils, but this phenomenon has not yet been confirmed in other plant species. Thus, it remains unclear to what extent barrier responses to nutrient limitation are nutrient-specific, species-specific, and shaped by local versus systemic regulation. Moreover, the shift in the progress of barrier differentiation has contradictory outputs to nutrient acquisition. Enhanced suberization of the barrier helps to prevent nutrient leakage from roots (Barberon et al., 2016; Vestenaa et al., 2024), but limits the radial transport of water and nutrients towards the stele. Differentiation of exodermis also restricts diffusion of nutrients into cortical apoplast and involvement of cortical membrane transporters in nutrient acquisition to symplast (Enstone et al., 2003).

This study is built on our previous study Namyslov et al. (2020), which showed that N and P deficiency enhanced exodermal differentiation in maize (*Z. mays)*, while K delayed it. It also documented that nutrient availability locally regulated endodermal and exodermal differentiation in the split-root system of maize.

The present study focused on the exodermal layer and extended the study of Namyslov et al. (2020) in two complementary directions. In the first part, it analyzes whether nutrient-induced changes in the progress of exodermal differentiation occur in other crop species and if these changes are common, variable, and locally-regulated. The panel of analyzed species included the following crops - sorghum (*Sorghum bicolor*), wheat (*Triticum aestivum*), barley (*Hordeum vulgare*), onion (*Allium cepa*), sunflower (*Helianthus annuus*), tomato (*Solanum lycopersicum*), flax (*Linum usitatissimum*), and pepper (*Capsicum annuum*), and the model species *Brachypodium distachyon*. The species were selected to represent phylogenetically diverse crops, known to form exodermis, enabling assessment of whether nutrient-dependent modulation of exodermal differentiation is conserved across taxa differing in root system architecture and ontogeny. To account for expected interspecific variability, we focused anatomical analyses on species-specific regions along the root axis corresponding to the zone of ongoing exodermal CB differentiation. Nutrient-related changes were then evaluated on a relative basis across species as change in the differentiation stage at this position. We tested deficiencies of four essential elements (N, P, K, and Fe) using standardized hydroponic systems and local response to N deficiency in split-root cultivation.

In the second part, maize (*Zea mays*) was used as a suitable model to analyze the functional and hormonal context of this response, as the local exodermal responsiveness of this species to nutrient stress has already been well described (Namyslov et al. 2020). We analyzed nutrient status, shoot performance, root apoplast permeability, and phytohormone levels in maize roots grown in split-root systems under uneven N or K supply. In addition, we tested the effects of selected exogenous phytohormones on exodermal development.

The objectives of the present study were: (i) to determine whether deficiencies of major nutrients, particularly N, commonly enhance exodermal differentiation across selected monocotyledonous and dicotyledonous species, (ii) to test whether local N deficiency induces local exodermal responses in split-root systems in other species than maize, and (iii) explore the functional consequences and hormonal context of local barrier modulation using maize as the model species. This design allowed us to combine broad interspecific verification of nutrient-induced exodermal responses with a more detailed analysis linking barrier differentiation to functional characteristics and phytohormone regulation in maize.

## Materials and methods

### Exodermal responses to homogeneously applied nutrient deficiencies in crop species

Plant material included monocotyledonous crops *Hordeum vulgare* L. cv. Golden Promise, *Sorghum bicolor* (L.) Moench cv. Ruzrok, *Triticum aestivum* L. cv. Anabel, *Allium cepa* L. cv. Vsetana, the monocot model species *Brachypodium distachyon* (L.) Beauv., and dicotyledonous crops *Capsicum annuum* L. cv. Slovakia, *Solanum lycopersicum* Mill. cv. Bajaja, *Helianthus annuus* L., and *Linum usitatissimum* L. Seeds were obtained from commercial suppliers (MoravoSeed CZ a.s., MoravoSeed Slovakia s.r.o., Osiva Moravia s.r.o.). All selected species were previously shown to be capable of differentiation of exodermal Casparian bands in the outer cortical layer (our observation and (Peterson et al., 1981; Perumalla et al., 1990; Xu et al., 2013; Bathoova et al., 2018; Namyslov et al., 2020; Sexauer et al., 2021)), although in *T. aestivum* and *H. vulgare* it is rather limited (Taleisnik et al., 1999; Ranathunge et al., 2017).

Cereal seeds and seeds of *B. distachyon* were germinated on moist filter paper in the dark at 25 °C for 2 days. Seeds of dicot species were germinated in a sterilized substrate (peat and silica sand mixture 2:1) in a growth chamber at 22 °C for 7–14 days. Nutrient deficiency effects were assessed in hydroponic cultivation under controlled growth conditions (16/8 h day/night photoperiod, 22/18 °C day/night temperature, 230 µmol m^-^² s^-^¹ PAR). Uniformly sized seedlings (and dormant *A. cepa* bulbs) were transferred to 12 L plastic containers (9 plants per container). The control solution consisted of quarter-strength modified Hoagland 3 solution, and four treatments induced deficiency of N, P, K, or Fe (**Table 1**). Plants were cultivated for 14 days, with complete solution replacement twice a week. Continuous aeration was provided, and the solution surface was light-protected with pieces of polyethylene foam (Mirelon, Mirel Vratimov, Czech Republic) to prevent algal growth.

**Table 1:** Composition of cultivation solution in experimental treatments.

Nutrients	Control treatment	-N treatment	-P treatment	-K treatment	-Fe treatment
Macronutrients (mM)					
Ca(NO3)2·4H2O	1.25	0	1.25	1.875	1.25
KNO3	1.25	0	1.25	0	1.25
KH2PO4	0.25	0.25	0	0	0.25
MgSO4.7H2O	0.25	0.25	0.25	0.25	0.25
CaCl2.2H2O	0	1.25	0	0	0
K2SO4	0	0.625	0.125	0	0
NaH2PO4·2H2O	0	0	0	0.25	0
Micronutrients (µM)					
Fe citrate	20	20	20	20	0
H3BO3	11.56	11.56	11.56	11.56	11.56
MnCl2·4H2O	2.29	2.29	2.29	2.29	2.29
ZnSO4	0.34	0.34	0.34	0.34	0.34
(NH4)6Mo7O24·4H2O	0.02	0.02	0.02	0.02	0.02
CuSO4	0.12	0.12	0.12	0.12	0.12

The length of the axis of the longest root, shoot length, and shoot fresh and dry weight (after drying to constant weight at 60 °C) were measured (*n* = 6-9) upon harvest. The longest root within root system was sampled for anatomical analysis to handle the variation of root system organization and root types. In dicots, this was the main primary root, whereas in monocots, where primary roots were absent (*A. cepa*) or indistinguishable after 14 days (cereal species), the longest roots generally corresponded to longest adventitious (nodal) roots.

### Localized exodermal responses to N deficiency in split-root cultivated crops

Localized root responses to N deficiency were evaluated using split-root hydroponics in species listed above. Seeds were germinated as described above, and seedlings were pre-cultivated in control solution to allow for sufficient root development and splitting. Pre-cultivation was performed under the same conditions described above.

In cereals, all roots were removed from germinated seedlings except two adventitious roots, and seedlings were pre-cultivated for two days. *A. cepa* bulbs were pre-cultivated for four days before retaining only two adventitious roots. Dicot seedlings were pre-cultivated for 7–14 days, depending on seedling growth rate, after which the main primary root was excised except for its basal part, allowing the split of equal-size halves of root system (lateral roots or adventitious roots emerging from the hypocotyl). Seedlings were then transferred to split-root containers (6 per container) with two separate 7 L chambers and cultivated for 14 days, with one chamber containing control solution (control side) and the other N-deficient solution (-N side). Growth conditions were as described above, and solutions were renewed once during cultivation.

The length of the longest root in each compartment of the split-root system was measured and selected for anatomical analysis upon harvest. In monocots, the longest adventitious root was sampled; in dicots, the longest root from the shoot base (adventitious root from the hypocotyl or lateral root from the primary root base) was used. Roots of similar size and comparable positions were consistently sampled from both nutritional compartments (*n* = 5-8). Additionally, in *L. usitatissimum*, the longest first-order lateral roots were collected from the longest adventitious root in each compartment of the split-root system to assess the response of the higher-order roots (*n* = 5).

### Root anatomical analyses

Roots were fixed in 4% formaldehyde. Exodermal differentiation was assessed by histochemical staining of 100 µm sections prepared with a hand microtome (Soukup, 2019). SL were detected using 0.1% (w/v) Sudan Red 7B (Brundrett et al., 1988; Soukup, 2019), while lignified CB were stained with 0.1% (w/v) berberine hemisulphate and counterstained with 0.05% (w/v) crystal violet (Brundrett et al., 1988; Soukup, 2019). All chemicals were obtained from Sigma-Aldrich, Inc. Sections were mounted in 65% aq. glycerol and imaged using an Olympus BX51 microscope equipped with a Nikon Digital Sight DS-U3 camera. SL were observed under bright field, and CB under UV excitation (U-MWU2 filter cube).

The presence of lignified CB was used as the anatomical marker of exodermal differentiation. Exodermal differentiation was evaluated semi-quantitatively based on the proportion of exodermal cells exhibiting detectable CB using the following scoring categories: 0, complete absence; 1, up to one-third of cells; 2, one-third to two-thirds of cells; 3, more than two-thirds of cells but not complete; and 4, complete presence in the exodermal layer. These categories reflect progression of exodermal differentiation into the primary developmental stage with lignified CB. The same scoring scheme was applied for exodermal and endodermal SL to quantify progression to the secondary suberized stage.

To approximate the position of exodermal differentiation along the root axis, a simplified initial analysis was first performed for each species using a single root from the N-deficient treatment. Cross sections were prepared at multiple positions along the root axis to identify the approximate beginning point of exodermal CB lignification. Based on this initial mapping, detailed anatomical analyses were subsequently conducted for all nutritional treatments using one species-specific relative position along the root axis (*n* = 4–7). Because roots were cultivated for the same period and exhibited comparable overall length within each species, tissues sampled at the selected position were assumed to represent a similar developmental stage across treatments. This approach was adopted to accommodate substantial interspecific differences in root anatomy and developmental patterning while maintaining a feasible sampling strategy capable of detecting treatment-induced shifts in exodermal differentiation. In species exhibiting only weak or delayed exodermis formation under the experimental conditions (*H. vulgare* and *T. aestivum*), analyses were performed at the most basal root segment (1–2 cm from the base).

The above-described sampling strategy was applied again independently, and based on separate root measurements, to roots obtained from split-root experiments.

### Split-root cultivation of *Zea mays* – plant performance, nutrient content, and root apoplast permeability

*Zea mays* cv. Cefran (Oseva Bzenec, Czech Republic) was cultivated in split-root hydroponics following Namyslov et al. (2020). Seeds were germinated on filter paper for 4 days at 25 °C, pre-cultivated in control solution for 2 days, and all roots except two adventitious roots were removed. These roots were distributed into split containers with two 7 L chambers. Treatments included N, P, and K deficiencies in control/control (C/C), control/deficient (C/–N, C/–P, C/–K), and deficient/deficient (–N/–N, –P/–P, –K/–K) setups. Cultivation lasted 10 days under the conditions described above, with aeration and a single solution renewal.

At harvest, the primary root axis length, shoot length, and fresh and dry shoot weight were measured. For elemental analysis, shoot and root biomass was homogenized with a Retsch MM301 mixer mill (Retsch, Germany). Potassium and phosphorus content was determined by flame atomic absorption spectrometry (SpectrAA 280 FS, Varian, USA) after HNO_3_/H_2_O_2_ digestion. Carbon (C) and N contents were analyzed using a Vario PYROcube elemental analyzer (Elementar Analysensysteme GmbH, Germany). Net photosynthetic rate (µmol CO_2_ m^-^² s^-^¹) and transpiration rate (mmol H_2_O m^-^² s^-^¹) were measured on intact plants using a LI-6400 portable gas analyzer (Li-COR, Lincoln, NE) at 23 °C, 450 ppm CO_2_, and 200 µmol m^-^² s^-^¹ PAR. Root apoplast permeability was assessed at the end of 10-days split-root cultivation by application of PTS (8-hydroxy-1,3,6-pyrenetrisulfonic acid trisodium salt; Acros Organic) apoplastic tracer according to Yeo et al. (1999). On the tenth day of cultivation, PTS (30 µM) was applied to the cultivation medium, followed by measurement of shoot accumulation after 24 h. Total shoot fresh biomass was extracted in 10 mL H_2_O at 90 °C for 2 h, and fluorescence of the extract (403 nm excitation, 510 nm emission) was measured on CLARIOstar 0430 (BMG Labtech) equipped with CLARIOstar software 5.40 and MARS data analysis software 3.32., and expressed as the difference from the respective negative controls without PTS application.

### Phytohormone analyses and exogenous applications in *Zea mays*

Phytohormone levels in maize roots were analyzed after 10 days of split-root cultivation (*n* = 3). Apical unbranched segments of the adventitious roots (from 1/6 of the root length to 1 cm behind the tip) from each half of the split-root systems were harvested and immediately frozen. Hormones and their metabolites were extracted and quantified using LC-MS/MS (Ultimate 3000, Dionex; 3200 Q TRAP) as described by Prerostova et al. (2020). Profiling included ABA and its metabolites, 1-aminocyclopropane-1-carboxylic acid (ACC), indole-3-acetic acid (IAA) and its derivatives, salicylic acid (SA), jasmonic acid (JA) and its derivatives, benzoic acid (BzA), *trans*-zeatin (tZ) and range of cytokinins derivatives; for full list of analyzed compounds see **Supplementary Table 1**.

For exogenous hormone treatments, maize seedlings (4 DAG) were grown hydroponically for 8 days (8 plants per 5.5 L container) in control solution, supplemented with ABA, cytokinin 6-benzylaminopurine (BAP), ethylene precursor ACC, or synthetic auxin 1-naphthaleneacetic acid (NAA). Phytohormones were applied at multiple concentration range (0-2000nM ABA, 0-1000nM ACC, 0-80nM NAA, and 0-4nM BAP), and primary root growth rate (cm day^-^¹) was monitored. Exodermal differentiation was evaluated histochemically, as described above, at the end of the cultivation period.

### Statistics

Statistical analyses were conducted using NCSS 9.0.15 (Hintze, 2013). Effects of nutrient treatments on apoplastic barrier development were assessed using the Kruskal-Wallis Z multiple-comparison test (whole-root hydroponics) or Wilcoxon Signed-Rank test (paired split-root data). Biometric traits were analyzed by one-way ANOVA followed by Tukey-Kramer multiple comparison test. Tests of assumptions involved Modified-Levene Equal-Variance test and D´Agostino Skewness, Kurtosis, and Omnibus tests. Data were ln-transformed when necessary.

## Results

### Interspecific differences in exodermal differentiation among crop species

**Figure 1:**
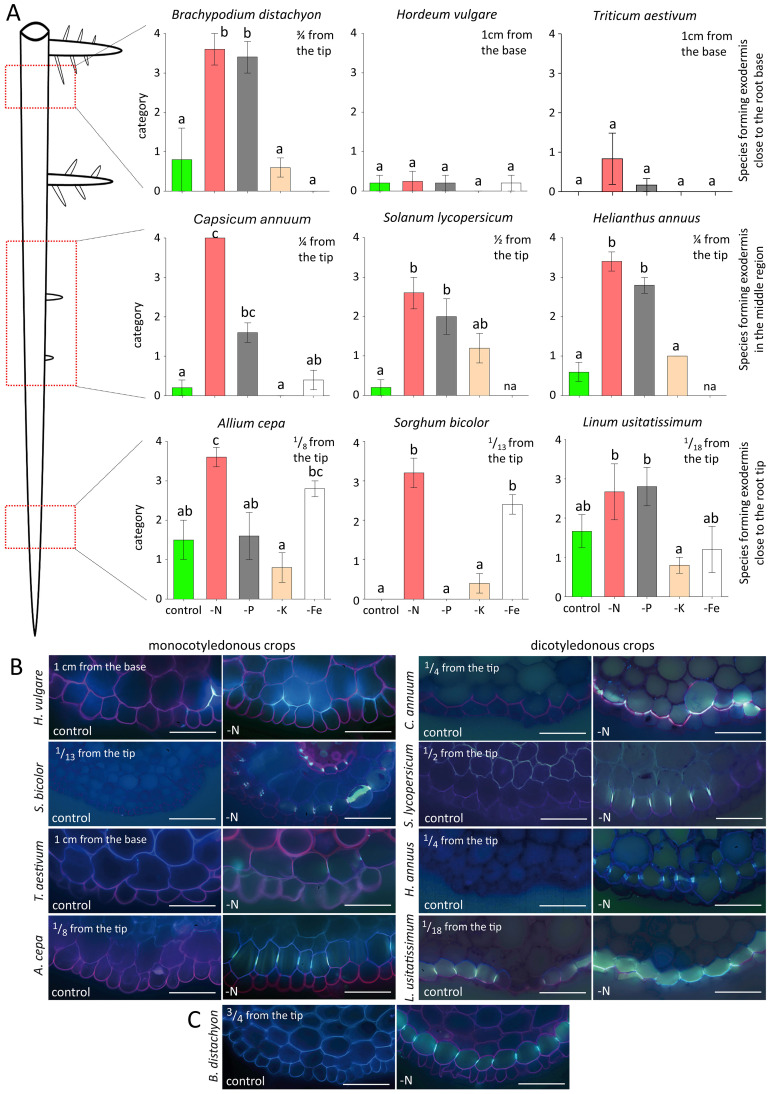
Differentiation of exodermal Casparian bands (CB) in N, P, K and Fe deficiency. **(A)** The incidence of cells with CB in the exodermal layer (mean ± SE). The category 0-4: 0 (complete absence), 1 (up to ⅓ of cells), 2 (⅓ to ⅔ of cells), 3 (⅔ of cells up to almost complete), and 4 (complete presence). Different letters indicate significant differences (Kruskal-Wallis Z multiple-comparison test, p<0.05, *n* = 4-7; na – data not available (not collected)). The upper right corner of each graph shows the relative position along the root axis where the root was sectioned for the treatment comparison in a given species. **(B, C)** Exodermal CB in monocotyledonous and dicotyledonous crop species **(B)** and *B. distachyon* **(C)** in control and N deficient treatments. The relative position along the root axis is indicated above photograph. Berberine hemisulphate + crystal violet staining for lignification (CB stained bright yellow), UV excitation, scale bars 50µm, representative images from 5 biological replicates.

The simplified initial anatomical analysis indicated differences in the position where exodermal CB can be detected behind the root apex of the longest root sampled from plants after 14 days of hydroponic cultivation (**Figure 1**). *L. usitatissimum*, *S. bicolor*, and *A. cepa* initiated formation of exodermal CB close to the root tip. *S. lycopersicum*, *H. annuus*, and *C. annuum* exhibited intermediate pattern, with exodermal CB detected around the middle zone of the main root axis. In *B. distachyon*, exodermal CB differentiation started substantially farther from the tip, with lignification of CB detected in more basal root regions. In *H. vulgare* and *T. aestivum*, exodermal CB differentiation was rather limited and detected in the very basal region. These findings suggested that the spatial progression of exodermal differentiation is species-specific and reflects inherent differences in root developmental programs.

In *S. lycopersicum*, *C. annuum*, and *L. usitatissimum*, exodermal lignification involved not only CB in radial cell walls but also outer tangential walls. In the present study, quantitative analyses focused exclusively on the presence of Casparian bands in radial walls, while tangential-wall lignification was not systematically evaluated.

### Exodermal differentiation is commonly enhanced by N deficiency in crop species

**Figure 2:**
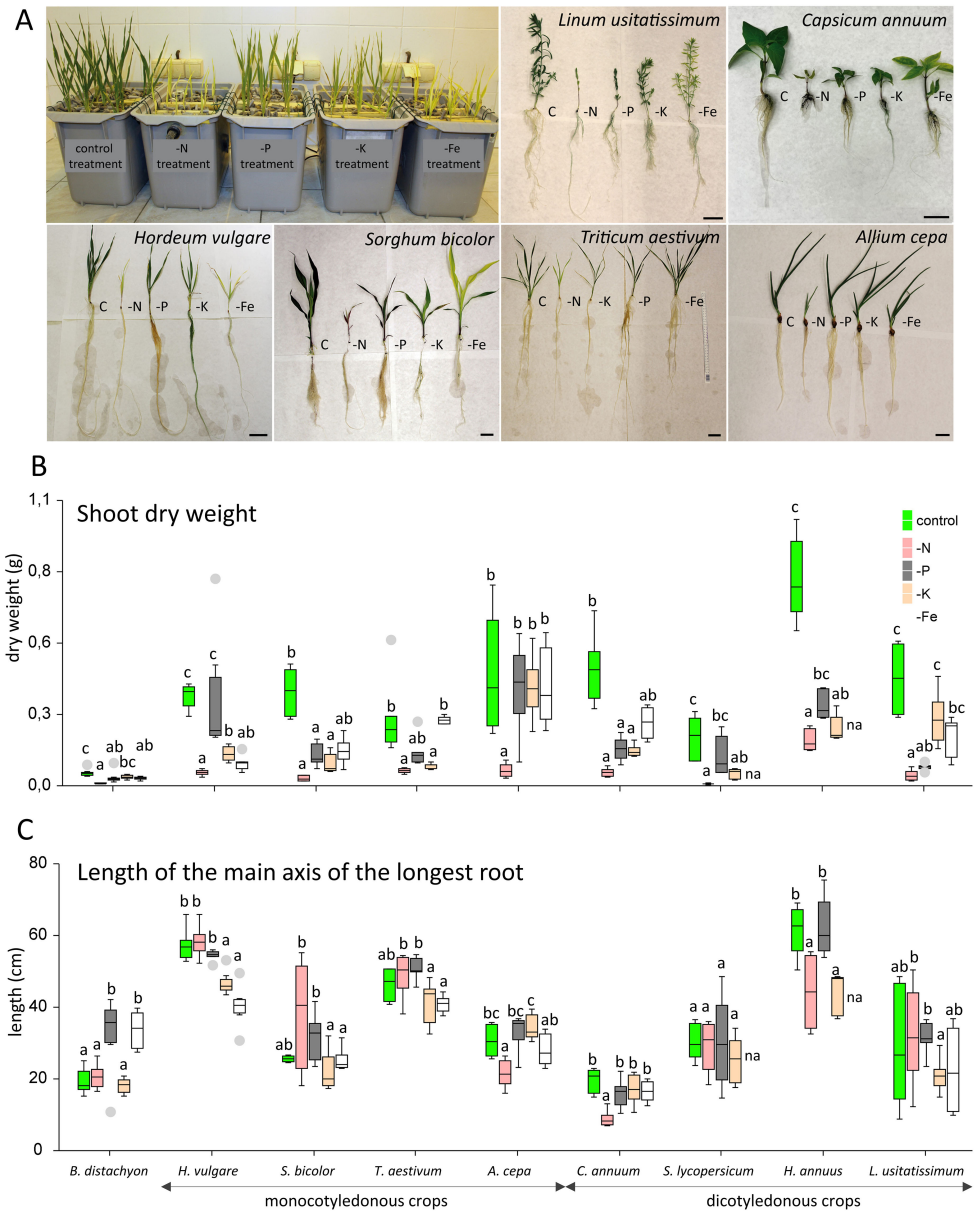
Growth of crop species in nutrient treatments. **(A)** Plant appearance after 14 days of hydroponic cultivation with N, P, K, and Fe deficiency. **(B)** Shoot dry weights (mean ± SE). **(C)** Lengths of the main axis of the longest roots (mean ± SE). Different letters indicate significant differences among treatments in the given species (Kruskal-Wallis Z multiple comparison test, p<0.05, *n* = 6-9). Scale bars 5 cm; na – data not available (not collected for technical reasons).

Among the applied nutrient deficiencies (N, P, K, Fe), N deficiency had the strongest effect on overall plant performance. In all analyzed crop species, as well as in *B. distachyon*, N-deficient plants exhibited markedly reduced shoot dry weights compared with controls and showed general leaf chlorosis (**Figures 2A, B**). Phosphorus and potassium deficiencies had generally milder effects on shoot biomass, which was nonetheless reduced relative to controls in some species (**Figure 2B**). Fe deficiency mostly did not cause the inhibition of shoot biomass production (**Figures 2A, B**). Visual symptoms of deficiency primarily included chlorosis (in N and Fe deficient plants) or drying of the leaf apices (in K deficient plants), however, the severity of the symptoms varied among species (**Figure 2A**).

The length of the main axis of the longest root within root system was reduced under N deficiency in *C. annuum*, and *H. annuus*, while no significant differences were observed in the remaining species (**Figures 2A,C**). Phosphorus deficiency did not affect this parameter in any species except *B. distachyon*, which developed longer roots under P deficiency than under control conditions. Potassium deficiency had only minor effects, reducing root axis length in *H. vulgare* and *H. annuus* (**Figures 2A, C**).

Nitrogen deficiency exerted the most pronounced and consistent stimulatory effect on exodermal differentiation in most species, which is consistent with our previous results for *Z. mays* (Namyslov et al., 2020). Exodermal CB formation was evaluated as described above to capture relative shift in the spatial onset of CB lignification. This approach provides a proxy for change in the proportion of root surface transitioning from apoplast-accessible to exodermis-restricted. Enhanced formation of exodermal CB under N deficiency was detected in *B. distachyon*, *S. bicolor*, *A. cepa*, *C. annuum*, *S. lycopersicum*, and *H. annuus* (**Figures 1A–C**). *L. usitatissimum* and *T. aestivum* showed similar trends that did not reach statistical significance (**Figures 1A, B**) and *H. vulgare* did not respond. Despite comparable root lengths across treatments (40–60 cm; **Figure 2C**), only a few lignified exodermal CB were detected 1 cm from the root base in *H. vulgare* in all the treatments (**Figures 1A, B**).

Phosphorus deficiency also stimulated lignification of exodermal CB, albeit to a lesser extent than N deficiency. This effect was statistically significant in four species (*B. distachyon*, *C. annuum*, *S. lycopersicum*, and *H. annuus*; **Figure 1A**). In contrast, K deficiency did not induce significant changes in exodermal CB formation in any species, while Fe deficiency had a stimulatory effect in *S. bicolor* (**Figure 1A**), underscoring the nutrient specificity of exodermal responses.

**Figure 3:**
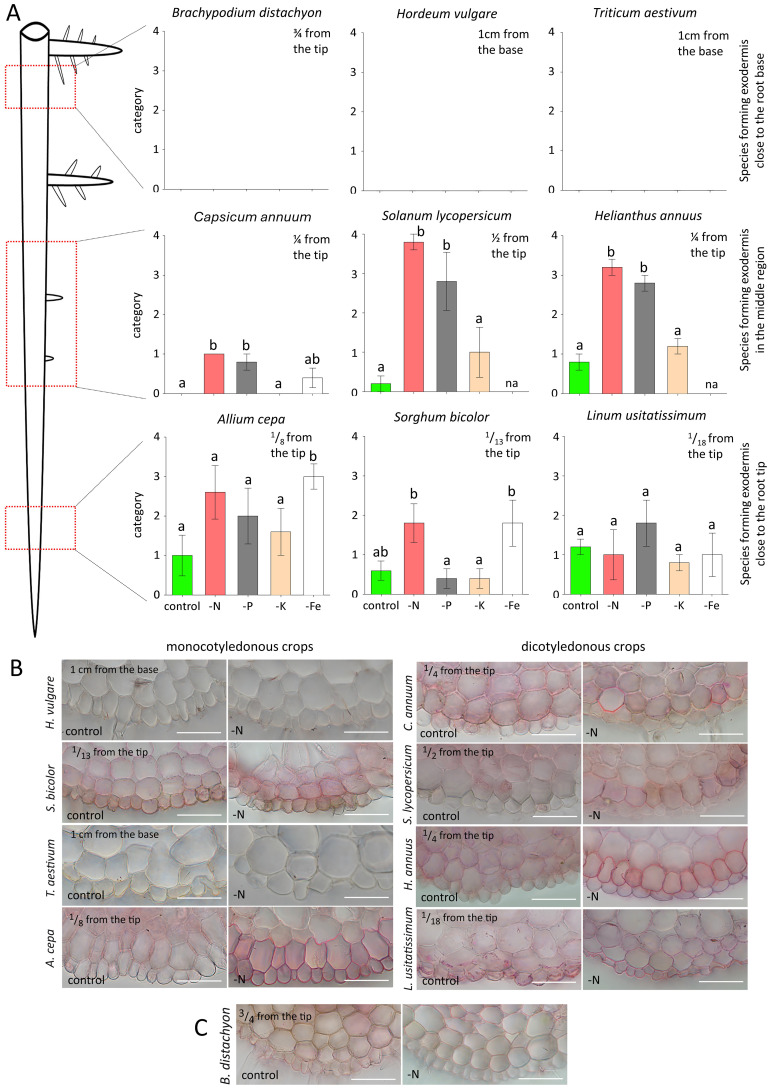
Deposition of exodermal suberin lamellae (SL) in N, P, K and Fe deficiency (mean ± SE). **(A)** The incidence of cells with SL in the exodermal layer. The category 0-4: 0 (complete absence), 1 (up to ⅓ of cells), 2 (⅓ to ⅔ of cells), 3 (⅔ of cells up to almost complete), and 4 (complete presence). Different letters indicate significant differences (Kruskal-Wallis Z multiple comparison test, p<0.05, *n* = 4-6). The upper right corner of each graph indicates the relative position along the root axis where the root was sectioned for the treatment comparison in a given species; na – data not available (data not collected for technical reasons). In *B. distachyon*, *H. vulgare*, and *T. aestivum* exodermal SL were not detected in any treatment, therefore graphs show zero values. **(B, C)** Exodermal SL in monocotyledonous and dicotyledonous crop species **(B)** and *B. distachyon* **(C)** in control and N deficient treatments. The relative position along the root axis is indicated above photographs. Sudan Red 7B, brightfield, scale bars 50µm. Suberin lamellae stained with Sudan Red 7B are visible as red-colored cell wall layer in exodermis.

In addition to CB, exodermal SL were scored at the same root positions as used for CB evaluation. Nitrogen deficiency enhanced exodermal suberization in *C. annuum*, *S. lycopersicum*, and *H. annuus* (**Figure 3**). Three species (*B. distachyon*, *T. aestivum*, *H. vulgare*) lacked detectable exodermal SL at the analyzed position, and three species (*A. cepa*, *S. bicolor*, *L. usitatissimum*) showed no significant response to N deficiency (**Figure 3**). Phosphorus deficiency enhanced exodermal suberization in *C. annuum*, *S. lycopersicum*, and *H. annuus*, while Fe deficiency induced this response in *A. cepa*. No changes were observed under K deficiency (**Figure 3**). Overall, patterns of suberin deposition largely mirrored those of CB formation, indicating coordinated regulation of both stages of exodermal differentiation under nutrient stress. For reference, the endodermal SL development in particular positions is shown in (**Supplementary Figure 1**).

### Locally driven exodermal responses to N deficiency in crop species

**Figure 4:**
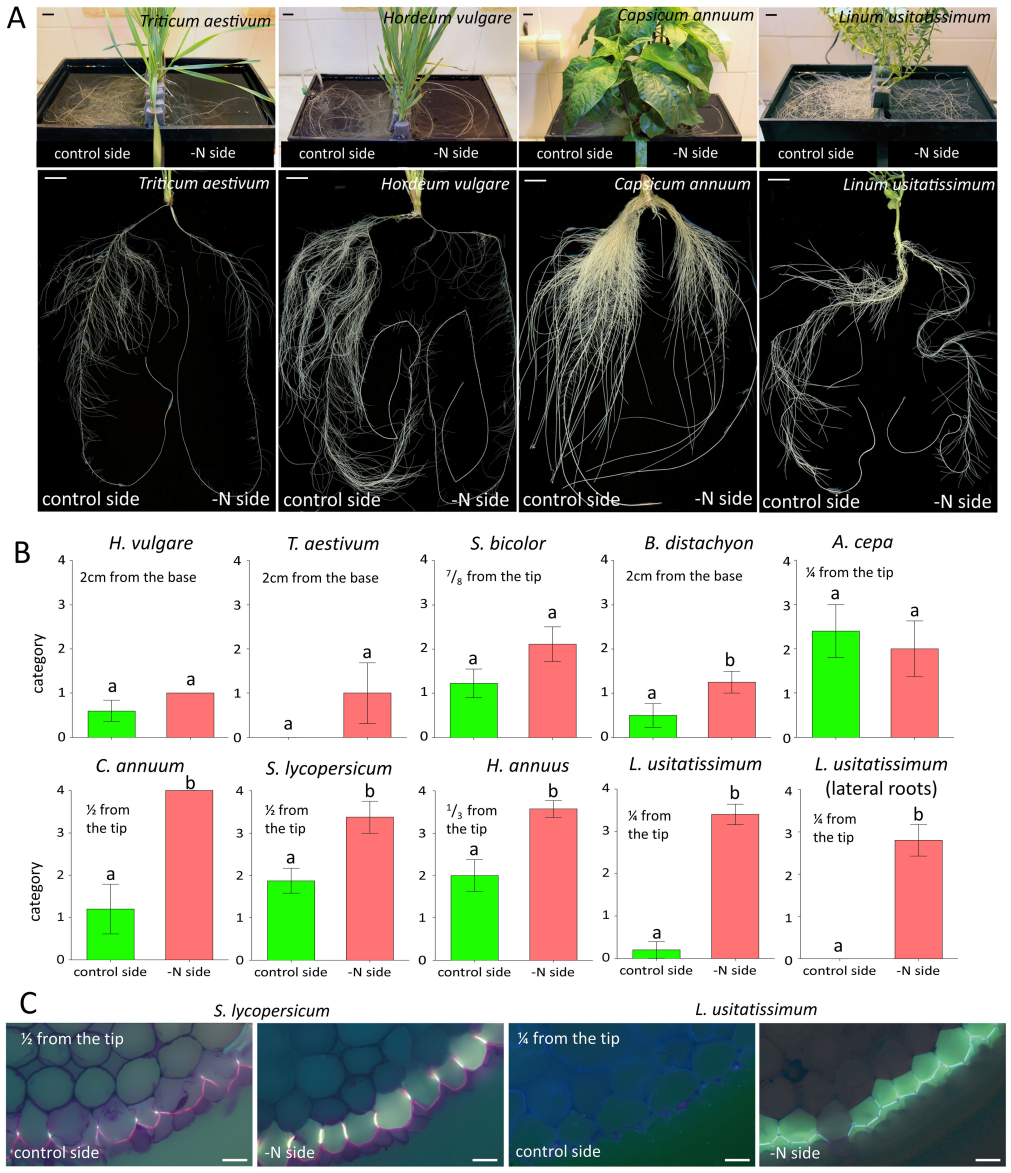
Local root responses to N deficiency in crop species in split-root hydroponics. **(A)** Shoot and root appearance at harvest. Scale bars 2 cm **(B)** The incidence of cells with CB in the exodermal layer (mean ± SE). The category 0-4: 0 (complete absence), 1 (up to ⅓ of cells), 2 (⅓ to ⅔ of cells), 3 (⅔ of cells up to almost complete), and 4 (complete presence). Different letters indicate significant differences (Wilcoxon Signed-Rank test p<0.05, *n* = 5-8). The relative positions along the root axis where roots were sectioned in each species are indicated in the graphs. **(C)** Exodermal CB in roots of *S. lycopersicum* and *Linum usitatissimum* in control and N-deficient side of split root systems. Berberine hemisulphate + crystal violet staining for lignification (CB stained bright yellow), UV excitation. The relative positions along the root axis are indicated above photographs. Scale bars 20 µm, representative images from 5 biological replicates.

Using split-root hydroponic system (**Figure 4A**), we tested whether exodermal response to N deficiency is locally regulated. Roots of all species were visibly more branched in the control compartment of the split-root system (**Figure 4A**). The length of the longest root axis generally did not differ between the two halves of the split-root system; exceptions were *B. distachyon* and *L. usitatissimum*, which developed longer roots on the N-deficient side, and *A. cepa*, which exhibited longer roots on the control side (**Supplementary Figure 2**).

Enhanced lignification of exodermal CB on the N-deficient side was observed in *B. distachyon* (**Figure 4B**) and all four dicotyledonous species (*C. annuum*, *S. lycopersicum*, *H. annuus*, *L. usitatissimum*; **Figures 4B, C**). *S. bicolor* showed the same trend, although closely missing statistical significance (p = 0.051; **Figure 4B**). *A. cepa* did not exhibit increased exodermal CB formation under local N deficiency.

In *H. vulgare* and *T. aestivum*, exodermal CB and SL were largely absent (undetectable by our histochemical approach) in the analyzed basal root region (2 cm from the root base) after 14 days of split-root cultivation. Consequently, it was not possible to assess whether these species exhibit localized exodermal response to nutrient deficiency. To gain at least some insight, we examined endodermis, even though it was not the primary focus of our study. The roots were analyzed closer to the tip, targeting the progression of endodermal suberization. Both species exhibited enhanced endodermal SL formation in N-deficient compartment compared with the control one (**Supplementary Figures 3A, B**), demonstrating their capacity for local endodermal adjustment in response to N deficiency. Therefore, we can conclude that the vast majority of species showed similar locally-driven exodermal (or endodermal) response to N deficiency as we previously found in split-root cultivated *Z. mays* (Namyslov et al., 2020).

Local responses were assessed also in lateral roots. The longest first-order laterals of split-root cultivated *L. usitatissimum* were analyzed. Exodermal differentiation was significantly enhanced in lateral roots from the N-deficient compartment compared with the control (**Figure 4B**), indicating that localized adaptation occurs both in main and lateral roots in flax and thus affecting a substantial portion of the absorptive root system.

### Effects of locally applied nutrient deficiency on nutrient contents, photosynthesis, transpiration and root transport properties in *Zea mays*

**Figure 5:**
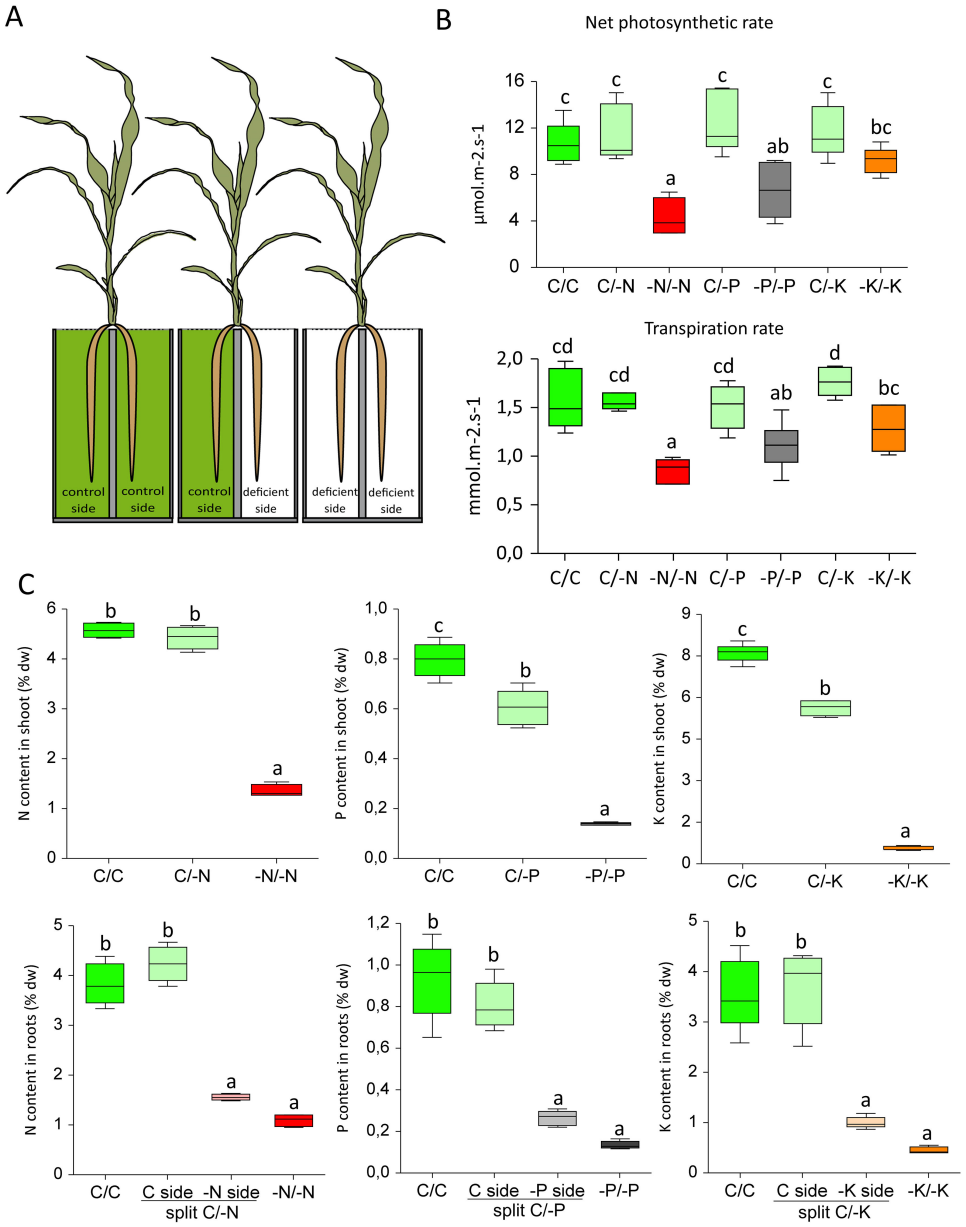
*Zea mays* in split-root cultivation. **(A)** Design of the split-root cultivation. **(B)** Net photosynthetic rate and transpiration rate. **(C)** Contents of the limiting nutrient (N, P, or K) in shoot and root biomass (mean ± SE). Plants were cultivated 10 days in split-root hydroponic cultivation with N, P or K deficiency in the following setup: control/control (C/C), control/deficient (C/–N, C/–K, and C/–P), and deficient/deficient (–N/–N, –K/–K, and –P/–P). Different letters indicate significant differences (Tukey-Kramer multiple comparison test, p<0.05, *n* = 5–6 in B, *n* = 4 in C).

*Z. mays* plants cultivated for 10 days in split-root hydroponic systems with locally applied N, P, or K deficiency (**Figure 5A**) exhibited net photosynthetic and transpiration rates comparable to control plants when only part of the root system was deficient (C/–N, C/–P, C/–K; **Figure 5B**). In contrast, plants subjected to complete N or P deficiency (–N/–N, –P/–P) showed significantly reduced photosynthesis and transpiration. In fully K-deficient plants (–K/–K), transpiration rates and net photosynthetic rates were not significantly different from controls (**Figure 5B**).

Nutrient analysis confirmed severe N, P, and K limitation in –N/–N, –P/–P, and –K/–K plants, respectively, and partial P and K limitation in C/–P and C/–K plants (**Figure 5C**). Shoot N contents were maintained in the C/–N treatment. In roots, nutrient concentrations in the control compartments of C/–N, C/–P, and C/–K split-root systems were similar to fully nourished controls, whereas deficient halves of root systems contained lower concentrations. These concentrations were slightly higher than in fully deficient roots (**Figure 5C**), suggesting partial retranslocation, but the differences were not statistically significant.

Apoplastic tracer assays using PTS revealed that roots from fully N-deficient plants (–N/–N) and from the N-deficient half of C/–N plants mediated significantly lower PTS transport than control roots and roots from the control half of C/–N plants (**Supplementary Figures 4A, B**), indicating reduced apoplastic permeability under N deficiency.

### Phytohormone profiles in roots of split-root cultivated *Zea mays*

**Figure 6:**
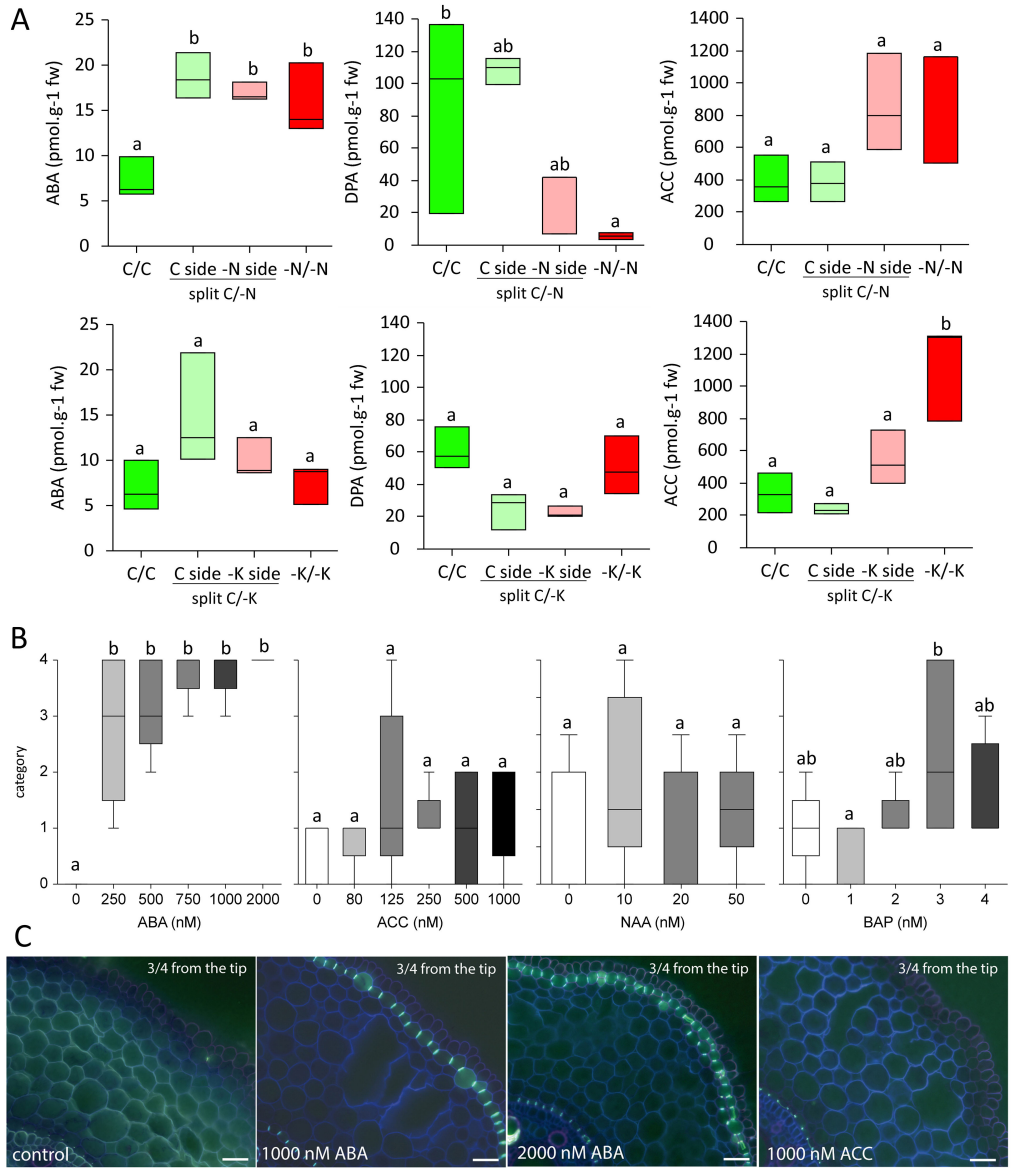
Internal phytohormone levels in *Z. mays* roots under locally applied deficiency and effects of phytohormone treatments on exodermal differentiation (mean ± SE). **(A)** ABA, DPA and ACC contents in roots of *Z. mays*, 10-day split-root hydroponics with N or K deficiency (control/control (C/C), control/deficient (C/–N, C/–K), and deficient/deficient (–N/–N, –K/–K)). Different letters indicate significant differences (Tukey-Kramer multiple comparison test, p<0.05, *n* = 3). **(B)** The incidence of cells with CB in exodermis in *Z. mays*, 8-day hydroponics, control solution supplemented with ABA, ACC, NAA or BAP (mean ± SE). The category 0-4: 0 (complete absence), 1 (up to ⅓ of cells), 2 (⅓ to ⅔ of cells), 3 (⅔ of cells up to almost complete), and 4 (complete presence). Roots were sectioned in the position ¾ of the length from the tip for treatment comparison. Different letters indicate significant differences (Kruskal-Wallis Z multiple-comparison test, p<0.05, *n* = 5). **(C)** Berberine hemisulphate + crystal violet staining for lignification (CB stained bright yellow), UV excitation. The relative positions along the root axis are indicated above photographs. Scale bars 50 µm, representative images from 5 biological replicates.

Phytohormones were analyzed in apical root segments after 10 days of split-root cultivation with locally applied N or K deficiency. Nitrogen deficiency significantly increased ABA concentrations in both fully (–N/–N) and partially (C/–N) deficient treatments. Elevated ABA levels were detected in both halves of the C/–N root system (**Figure 6A**; **Supplementary Table 1**). Levels of DPA, an inactive ABA catabolite, were reduced in N-deficient roots, resulting in an increased ABA/DPA ratio. ACC concentrations tended to be higher in N-deficient roots (p = 0.055; **Figure 6A**; **Supplementary Table 1**). Other phytohormones and metabolites showed no consistent differences, except for significantly higher indole-3-acetic acid in the N-deficient roots and in both halves of the C/-N split-root system (**Supplementary Table 1**). Moreover, we did not detect *trans*-zeatin in fully deficient treatment (-N/-N), suggesting that its concentration was below the detection limit, compared to other experimental treatments (**Supplementary Table 1**). Finally, jasmonic acid was elevated in both halves of the C/-N split-root system (**Supplementary Table 1**).

Potassium deficiency did not significantly affect ABA but increased ACC in fully K-deficient roots (–K/–K), indicating enhanced ethylene biosynthesis. Roots from the K-deficient half of C/–K systems showed a similar but weaker trend. Fully K-deficient roots also accumulated higher levels of some cytokinin metabolites. These changes were absent in roots from C/–K treatments. Overall, no phytohormone consistently distinguished control and deficient compartments under K deficiency (**Supplementary Table 1**). In addition, correlation analysis did not reveal any consistent correlations between phytohormone levels in roots and the state of exodermal and endodermal differentiation in roots that would be present in both split-root nutrient treatments (**Supplementary Table 1**).

### Effects of phytohormone application on exodermal differentiation in *Zea mays*

**Figure 7:**
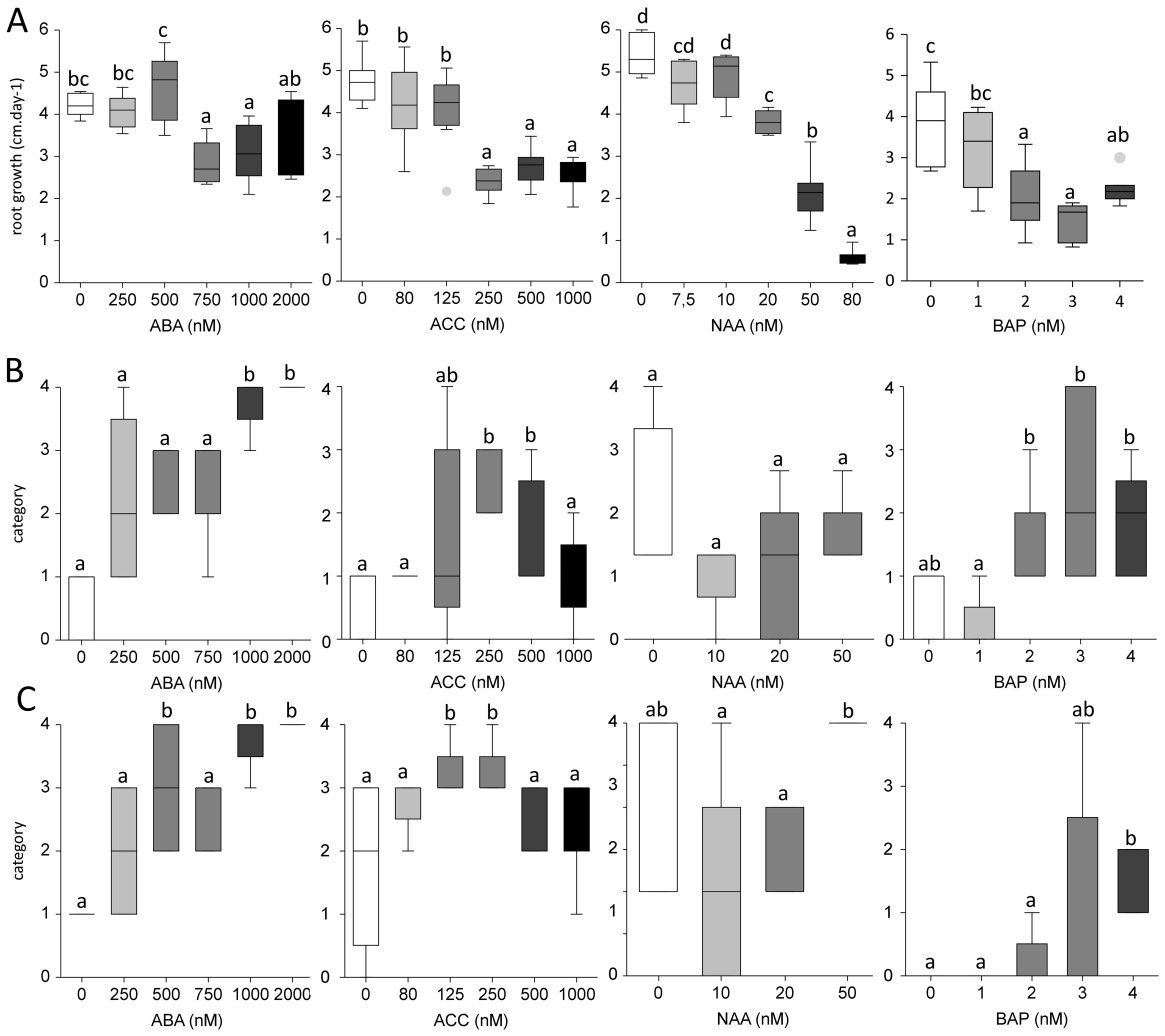
Phytohormone effects on root growth and suberin lamellae (SL) deposition in *Z. mays*. **(A)** Root growth (average daily increment of the primary root main axis in cm.day-1) in *Z. mays*, 8-day hydroponics, control solution supplemented with ABA, ACC, NAA or BAP (mean ± SE). Different letters indicate significant differences (Tukey-Kramer multiple comparison test, p<0.05, *n* = 7-8). **(B, C)** The incidence of cells with SL in exodermis **(B)** and endodermis **(C)**. The category 0-4: 0 (complete absence), 1 (up to ⅓ of cells), 2 (⅓ to ⅔ of cells), 3 (⅔ of cells up to almost complete), and 4 (complete presence). Roots were sectioned in the position ¾ of the length from the tip for treatment comparison. Different letters indicate significant differences (Kruskal-Wallis Z multiple comparison test, p<0.05, *n* = 5, mean ± SE).

*Z. mays* plants were cultivated hydroponically for 8 days in control nutrient solution supplemented with ABA, ACC, NAA, or BAP. ABA strongly stimulated exodermal differentiation, inducing early lignification of CB in a concentration-dependent manner (**Figures 6B, C**). Effects were detectable at 500 nM ABA, which did not inhibit root elongation (**Figure 7A**). Concentrations ≥1000 nM induced lignification in two outer cortical layers (**Figure 6C**), representing an atypical phenotype not observed under standard growth conditions. ABA also enhanced suberization of exodermal and endodermal layers at higher concentrations (**Figures 7B, C**).

Cytokinin (BAP) promoted exodermal CB lignification, but only at high concentrations that inhibited root growth (**Figures 6B**, **7A**). ACC and NAA did not significantly affect exodermal CB formation at any tested concentration (**Figures 6B, C**) and reduced root elongation at higher concentrations (≥250 nM for ACC; ≥20 nM for NAA; **Figure 7A**). At elevated concentrations, ACC and BAP stimulated exodermal and endodermal suberization (**Figures 7B, C**). Collectively, ABA is identified as the primary positive regulator of exodermal differentiation in *Z. mays*, capable of inducing pronounced anatomical modifications of the outer cortex when applied exogenously.

## Discussion

### Exodermal responses to N and P deficiencies are widespread in crop species but modulated by species and genotype

Our comparative survey demonstrates that N deficiency, and, to a lesser extent, P deficiency, consistently accelerates exodermal differentiation across a broad range of crop species as well as in the monocot model *B. distachyon*. This response was observed as more pronounced formation of exodermal Casparian bands and/or suberin lamellae, indicating reinforcement of the cortical apoplast. These findings align with previous reports in *Z. mays*, where both homogeneous and locally applied N deficiency enhanced exodermal differentiation (Namyslov et al., 2020; Chen et al., 2023), but contrast with delayed barrier development reported in *R. communis* under comparable conditions (Schreiber et al., 2005). These data suggest that while N deficiency-induced acceleration of barrier differentiation is common, its expression is shaped by species-specific developmental programs.

An exception was *H. vulgare*, in which homogeneous N deficiency did not enhance exodermal differentiation in the cultivar used (cv. Golden Promise). Golden Promise is a historical spring barley variety that is used for malting and, more importantly, is highly genetically transformable, making it an important genotype for molecular studies of cereal crops (Schreiber et al., 2020). Barley is known to exhibit weak or delayed formation of exodermis under hydroponic conditions (Ranathunge et al., 2017), which we also observed in our 14-day-old plants. Importantly, this does not reflect a general inability of barley to adjust apoplastic barriers under nutrient stress. Other cultivars, such as cv. Henriette, formed a well-developed exodermis with CB and SL and responded to P deficiency with accelerated differentiation (Namyslov et al., 2020) and cv. Quench exhibited enhanced endodermal suberization under N and P limitation (Armand et al., 2019). These observations highlight intraspecific variation in barley.

It should be noted that N deficiency also caused the most pronounced inhibition of shoot growth across all species tested. The severity of deficiency may affect the response of the barrier. This has been documented for endodermal suberization in barley under manganese (Mn) deficiency (Chen et al., 2019) and it emphasizes the importance of considering the extent of nutrient limitation when interpreting the exodermal responses observed in our study. On the other hand, K deficiency significantly inhibited shoot growth in 6 out of 9 species, while no significant exodermal response was observed. Severity of deficiency may thus influence the extent of barrier response, but it is certainly not the only factor.

### Multiple patterns of exodermal lignification can occur in plants

In several dicot species (*S. lycopersicum*, *C. annuum*, *L. usitatissimum*), we observed lignification of the outer tangential wall of exodermal cells, occurring with variable frequency and intensity. This pattern closely resembles the “polar lignin cap” recently described in tomato exodermis, which functions as an effective apoplastic barrier and is under distinct genetic control from endodermal Casparian bands (Manzano et al., 2024). In tomato cv. M82, this polar lignin cap was present in exodermis without the co-occurrence of the typical Casparian bands in the radial walls (Manzano et al., 2024). By contrast, in the tomato cultivar examined in our study, lignification was more commonly present in radial walls typical of Casparian bands and only less frequently involved the outer tangential walls, particularly under N or P deficiency. This discrepancy indicates the existence of two separable exodermal lignification programs, which may reflect intraspecific variation among tomato cultivars or may be variably activated in response to different environmental cues. The presence of tangential-wall lignification in *C. annuum* and *L. usitatissimum* further suggests that this trait is not restricted to tomato but is more broadly distributed among dicots.

### Local enhancement of exodermal differentiation under N deficiency is a common adaptive strategy

Soil nutrient availability is highly heterogeneous in space and time, and roots have evolved numerous locally regulated mechanisms to exploit nutrient-rich patches (Drew, 1975; Lima et al., 2010; Zhang et al., 2014; Ruffel and Gojon, 2017), including localized root proliferation, local induction of high affinity nutrient transporters, and locally enhanced rhizosphere modifications, with particularly strong response to N, one of the most frequently limiting nutrients. Our results indicate that local modulation of apoplastic barriers can be added to this suite of adaptive mechanisms. Our split-root experiments demonstrate that locally enhanced exodermal differentiation under N deficiency is widespread among crop species. Faster formation of exodermal CB was detected specifically in root compartments exposed to N deficiency in four out of eight tested crops and in *B. distachyon*, consistent with our earlier findings in *Z. mays* (Namyslov et al., 2020). Importantly, the analysis of *L. usitatissimum* showed that this response is extended to lateral roots, indicating that localized regulation of apoplastic barriers can substantially influence transport properties at the scale of whole root system.

Even in species with weak formation of exodermis under our experimental conditions, such as *T. aestivum* and *H. vulgare*, localized responses were evident through enhanced endodermal suberization. This suggests that local modulation of cortical apoplastic permeability, whether via exodermis or endodermis, constitutes a general adaptive strategy. Although nutrient gradients in experimental split-root systems are more stable than those in natural soils, spatial gradients of nitrate and ammonium are well documented in both natural and agricultural environments (Cain et al., 1999; Zebarth et al., 1999; Rogers and Benfey, 2015), particularly under localized fertilization regimes. Consequently, local regulation of barrier differentiation is likely to be ecologically and agronomically relevant.

### K and Fe deficiencies elicit weak or inconsistent exodermal responses

In contrast to N and P deficiencies, K deficiency did not accelerate exodermal differentiation in any species examined. This adds to previously described delays in exodermal and endodermal differentiation reported in *Z. mays* and *H. vulgare* (Coffey et al., 2018; Namyslov et al., 2020) and to enhanced endodermal suberization in *A. thaliana* (Barberon et al., 2016). Fe deficiency likewise produced only minor effects in our study. The only significant effect was faster formation of exodermal CB in *S. bicolor* and enhanced deposition of exodermal SL in *A. cepa*, which contrasts with delayed endodermal suberization reported previously in *A. thaliana* (Barberon et al., 2016).

These contrasting responses cannot be explained solely by differences in nutrient mobility in soil or within plant tissues. Potassium is highly mobile most prevalent monovalent cation in cytoplasm. Nitrogen and phosphorus are also characterized by a high degree of mobility within plant tissues, allowing them to be readily translocated throughout the plant body, whereas Fe is rather immobile (Marschner, 2011). In soil, K and NO_3_^-^ are mobile and prone to leaching. However, the majority of K in soil is bound in minerals with limited release. Phosphorus mobility is generally very low and Fe is poorly soluble under neutral to alkaline conditions (Marschner, 2011). Yet only N and P deficiency consistently enhanced exodermal differentiation. Potassium deficiency did not accelerate exodermal differentiation in any of the species examined here, despite the high mobility of K^+^ in both plant tissues and soil, and despite evidence that endodermal suberization can effectively restrict K^+^ leakage from the stele in barley roots (Vestenaa et al., 2024). Experimental findings obtained using *A. thaliana* showed that proper endodermal lignification and suberization are important for maintaining plant K homeostasis, particularly under conditions of low relative humidity (Baxter et al., 2009; Pfister et al., 2014; Reyt et al., 2021). Similar roles can be assumed for the exodermis but its developmental response to K deficiency is still a puzzling phenomenon.

It is also fair to mention, that we have compared the extent of exodermal differentiation between nutrient treatments at one relative position along the root axis in each species. This approach allowed us to detect the enhancement of differentiation compared to control treatment very well, and such acceleration was not found in any of the analyzed species in response to K deficiency. However, we cannot fully exclude some underestimation of a possible suppression of differentiation.

### Physiological relevance of locally enhanced barriers

Nitrogen and phosphorus deficiency have been repeatedly associated with reduced root hydraulic conductivity (Carvajal et al., 1996; Gloser et al., 2007; Gorska et al., 2008; Coffey et al., 2018). In our study, we tested root apoplast permeability in split-root *Z. mays* using PTS apoplastic tracer. Although PTS has limitations to mimic water movement in roots (Steudle, 2000), it was repeatedly used to test root apoplast permeability (Peterson and Emanuel, 1981; Yeo et al., 1999; Faiyue et al., 2010; Pecková et al., 2016). The PTS test showed that roots growing in N-deficient compartment of split-root chamber mediated significantly smaller PTS accumulation in shoot compared to accumulation mediated by roots in the control compartment, which can be attributed to uneven differentiation of barriers as well as proliferation of lateral roots, as reported earlier from the split-root experiments in *Z. mays* in Namyslov et al. (2020). This result indicates that inhibition of root conductivity does not only prioritize root growth over shoot as suggested by Armand et al. (2019), but it may also help to target water uptake to nutrient-rich zones, a mechanism previously demonstrated e.g. in split-root systems of cucumber (Gorska et al., 2008). In agreement, *Z. mays* plants supplied with either N, P or K to only one half of root system in split-root cultivation chamber exhibited similar photosynthetic and transpiration rates compared to fully control plants, which indicates no inhibition of these processes, in contrast to fully deficient plants.

Barrier reinforcement may also reduce nutrient leakage from roots into nutrient-poor surroundings, as proposed for endodermal suberization (Barberon et al., 2016; Vestenaa et al., 2024). Slightly higher content of a limiting nutrient, which we detected in the deficient half of the split-root system of maize, compared to the fully deficient roots, indicates a partial retranslocation within the split-root system for N, P as well as K. This supports the importance of leakage prevention from roots growing in nutrient-poor soil patches. However, it is not clear what proportion of the retranslocated nutrient can be lost to the rhizosphere in the deficient part of split-root system, as we did not directly measure the leakage from the roots. Moreover, the observed difference in nutrient contents between halves of split-root system was relatively mild and not statistically significant. The absence of accelerated barrier formation under K deficiency also suggests that leakage prevention alone does not fully account for nutrient-specific exodermal responses, although suberization was proved to decrease K^+^ leakage across endodermal layer of barley (Vestenaa et al., 2024) and leakage of solutes and water across endodermis of *A. thaliana* (Wang et al., 2019).

### Phytohormonal regulation of exodermal differentiation in maize

Abscisic acid has emerged as a positive regulator of apoplastic barrier formation across multiple species (Barberon et al., 2016; Shiono et al., 2021; Shukla et al., 2021; Cantó-Pastor et al., 2024). Our results extend this role to *Z. mays*, in which exogenous ABA strongly promoted exodermal and endodermal differentiation.

However, endogenous ABA levels did not fully explain localized barrier responses under uneven N supply in maize roots. N-deficient roots of maize exhibited elevated ABA levels compared to fully-nourished controls, but we detected same high levels in both halves of split-root systems in C/-N treatment. This suggests that local sensitivity, transport, or perception of ABA, rather than absolute hormone levels, determine barrier development. It has been recently shown, that the transcriptional response to ABA differs based on nitrate availability, being suppressed under high-nitrate conditions but substantially increased under low-nitrate conditions, suggesting a tight integration of ABA signaling with nutrient availability (Ma et al., 2025). Specific transporters of nitrate transporter1/peptide transporter (NPF) family mediate ABA delivery to the endodermis (Binenbaum et al., 2023), and nitrate and ABA competitively bind to the nitrate transporter/sensor NRT1.1B, thereby integrating nutrient status with stress signaling (Ma et al., 2025). Moreover, at low nitrate, the complex of Calcineurin B‐like protein 1/9 (CBL1/9) and CBL-interacting protein kinase 23 (CIPK23) phosphorylates NRT1.1 at Thr_101_ increasing its affinity to nitrate, which promotes N uptake. However, the sucrose non-fermenting1 (SNF1)‐related protein kinase 2 (SnRK2.6), involved in ABA signaling, was found to phosphorylate NRT1.1 at position Ser_585_ inhibiting its nitrate transport activity in low nitrate surrounding (Su et al., 2021), which can occur, e.g., at -N side of the split-root system or in the case of uneven N distribution in the soil. At moderate nitrate levels, ABA has stimulatory effect on lateral root growth (Signora et al., 2001). Thus, our finding of elevated ABA levels both in complete N-deficiency as well as in both halves of the split-root system may be related to multiple ABA functions, which may differ based on the actual N concentration in the tissue.

Ethylene showed no consistent relationship with exodermal differentiation in maize, highlighting species-specific differences in hormonal control. Ethylene has been associated with delayed suberization of barriers in *A. thaliana* and *S. alfredii* (Barberon et al., 2016; Liu et al., 2020), associated with adaptive responses to changes in nutrient availability (Ahmad et al., 2023), and repeatedly linked with low-K stress signaling (Shin and Schachtman, 2004; Jung et al., 2009; Schachtman, 2015). In agreement, we detected high ACC levels in K-deficient roots of *Z. mays*, which might have slowed down exodermal differentiation under K deficiency. However, N-deficient roots had also relatively high ACC levels, although developmental response of barriers to N deficiency was the opposite. Moreover, ACC treatment did not delay exodermal differentiation in *Z. mays* roots. These data suggest that ethylene levels are not always correlated with the progress of barrier differentiation, and we can speculate that the effects of ethylene are likely context-dependent. Among others, ethylene levels in roots rise under hypoxia to trigger aerenchyma formation (Drew et al., 2000). In waterlogged hypoxic soil, fast differentiation of suberized exodermis is equally important, protecting the root from reduced toxic substances and radial oxygen loss (Armstrong et al., 2000; Soukup et al., 2007). In such case, ethylene inhibitory effect on suberization seems undesirable, which opens a new interesting direction for future research.

Another phytohormone associated with nitrate availability is auxin. Our finding of enhanced IAA level at N deficiency (-N/-N treatment) is in accordance with the result of Ma et al. (2014), who reported up-regulation of auxin biosynthesis gene *Tryptophan aminotransferase related 2 (TAR2*) in *A. thaliana* under low N conditions. Cytokinins also play a key role in communicating N availability between plant organs and regulating N partitioning and plant development (Gawronska et al., 2003; Sakakibara, 2003). Nitrogen stimulates the expression of the cytokinin biosynthetic gene isopentenyl transferase (Kamada-Nobusada et al., 2013), which is in accordance with detected up-regulation of the level of cytokinin precursor isopentenyladenosine phosphate in the split-root variant in +N part (C side). In our experiment, N deficiency reduced levels of active cytokinin *trans*-zeatin below the detection limit of the analysis, while split-root treatments increased its level in both parts of the root system, supporting its role in integrating N signals across the plant (Sakakibara, 2003). Application of cytokinin BAP into growth media inhibited root elongation and simultaneously promoted exodermal differentiation in *Z. mays*. Similar inhibition of root elongation was triggered by synthetic auxin NAA, but without the effect on exodermal layer.

Our results are also in partial accordance with Kamali and Singh (2022), who reported stimulation of jasmonate biosynthesis upon N deficiency. Recently, several members of the JA biosynthesis pathway were found to be induced during early and late N deficiency in *O. sativa* (Deepika and Singh, 2021). Moreover, ethylene-responsive transcription factor 109 (ERF109), induced by low N, was found to mediate crosstalk between JA and auxin biosynthesis, which suggests the synergistic action of JA and auxin in root system architecture modulation under N starvation (Cai et al., 2014; Lv et al., 2021). This accords with our results on elevation of immediate ethylene precursor ACC under N-deficiency (both at -N/-N and at -N side of the split root system).

Overall, our data indicate that barrier differentiation results from the integration of nutrient sensing, hormonal signaling networks, and local transport dynamics, rather than from changes in a single hormonal pathway.

### Do dicots differ from monocots in exodermal responses to deficiency?

In dicots, the development of the periderm renders the barrier function of exodermis and endodermis transient (Campilho et al., 2020). This may influence the dynamics of exodermal responses to nutrient deficiency in apical root regions, observed in dicots and monocots (Barberon et al., 2016; Armand et al., 2019; Liu et al., 2020; Namyslov et al., 2020).

Our short-term hydroponic experiments indicate that cereal species (Poaceae) generally initiate lignification of exodermal CB farther from the root apex than *A. cepa* (Amaryllidaceae) and all the dicot species from Solanaceae and Linaceae included in this study. Since exceptions occurred, such as CB formation close to the apex under N deficiency in *S. bicolor*, a consistent overall generalized pattern was not observed. It also has to be emphasized that we used rather simplified methodological approach to detect positions of exodermal CB lignification along root axis, and that plants grown in short-term hydroponic cultivations were analyzed. For these reasons, the obtained positions along root axis cannot be simply extrapolated to plants growing in soil or considered a general characteristic of the species.

The more distal onset of exodermal differentiation in cereals coincided with a generally weaker exodermal response to nutrient deficiency compared with dicots. However, the direction of the response was conserved across all species: N deficiency consistently acted as the strongest inducer of exodermal differentiation in both phylogenetic groups. Importantly, this trend remained robust despite substantial interspecific variation, including differences in the root types analyzed (e.g. seminal roots versus adventitious roots). Such differences may contribute to variability in absolute responses, as adventitious roots in cereals were shown to exhibit lower levels of suberization than seminal roots in *H. vulgare* (Vestenaa et al., 2024), but they do not alter the overall response pattern.

## Conclusion

Our survey of crop species demonstrates that the exodermis, an apoplastic barrier in the peripheral root cortex, is broadly responsive to nutrient stress across phylogenetically diverse crops. Although species differed in the spatial pattern of exodermal differentiation along the root axis, N deficiency consistently promoted the formation of Casparian bands and suberin lamellae in both monocots and dicots, irrespective of whether nutrient supply was uniformly or locally restricted. Phosphorus deficiency induced qualitatively similar but generally weaker responses, whereas K and Fe deficiencies produced only limited or inconsistent effects. These findings indicate that reinforcement of cortical apoplastic barriers represents a widespread and highly conserved response to nutrient limitation, particularly under N deficiency.

Our split-root experiments further revealed that localized enhancement of exodermal differentiation under heterogeneous N supply occurs across diverse crop species and extends to lateral roots. This supports the hypothesis that exodermal barrier formation may contribute to the local regulation of water and solute transport within complex root systems.

It should be noted that the experiments were conducted under controlled hydroponic conditions and focused primarily on anatomical responses. Future studies should therefore investigate how exodermal differentiation influences root system efficiency under environmentally more realistic conditions. In addition, N deficiency caused the strongest inhibition of shoot growth across all examined species, highlighting the importance of considering the severity of nutrient limitation when interpreting exodermal responses, as the magnitude of stress may directly influence the extent of barrier formation.

Overall, our results establish exodermal differentiation as a dynamic and nutrient-responsive component of root adaptation that integrates both systemic and local responses to nutrient limitation.

## Data Availability

The datasets presented in this study can be found in the Zenodo repository, https://doi.org/10.5281/zenodo.18481952. The names of the repository/repositories and accession number(s) can be found in the article/**Supplementary Material**.
