# Supplementary material for: Local adjustment of exodermis to nutrient deficiency in crop species
Source: Front Plant Sci. 2026 Jul 20;17:1817677. doi: 10.3389/fpls.2026.1817677 (PMC13429614; doi:10.3389/fpls.2026.1817677)
Supplement: Supplementary file 1 [file Table1.docx]

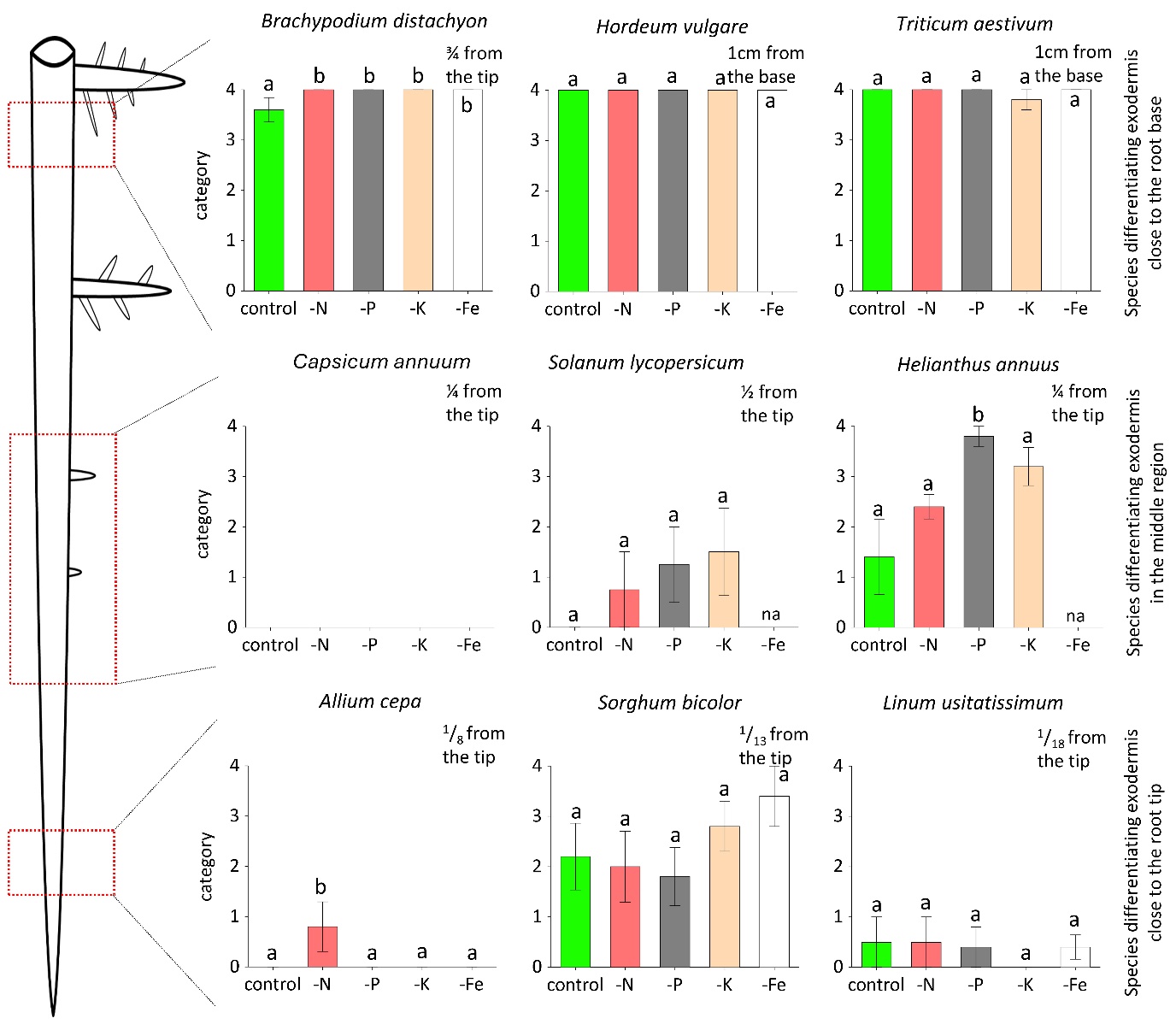


Supplementary Figure 1. Deposition of endodermal suberin lamellae (SL) in N, P, K and Fe deficiency (mean ± SE). **(A)** The incidence of cells with SL in the endodermal layer. The category 0-4: 0 (complete absence), 1 (up to ⅓ of cells), 2 (⅓ to ⅔ of cells), 3 (⅔ of cells up to almost complete), and 4 (complete presence). Different letters indicate significant differences (Kruskal-Wallis Z multiple comparison test, p<0.05, *n*=4-6). The upper right corner of each graph indicates the relative position along the root axis where the root was sectioned for the treatment comparison in a given species; na – data not available (data not collected for technical reasons). In *C. annuum* endodermal SL were not detected in any treatment at the given position, therefore graph shows zero values.


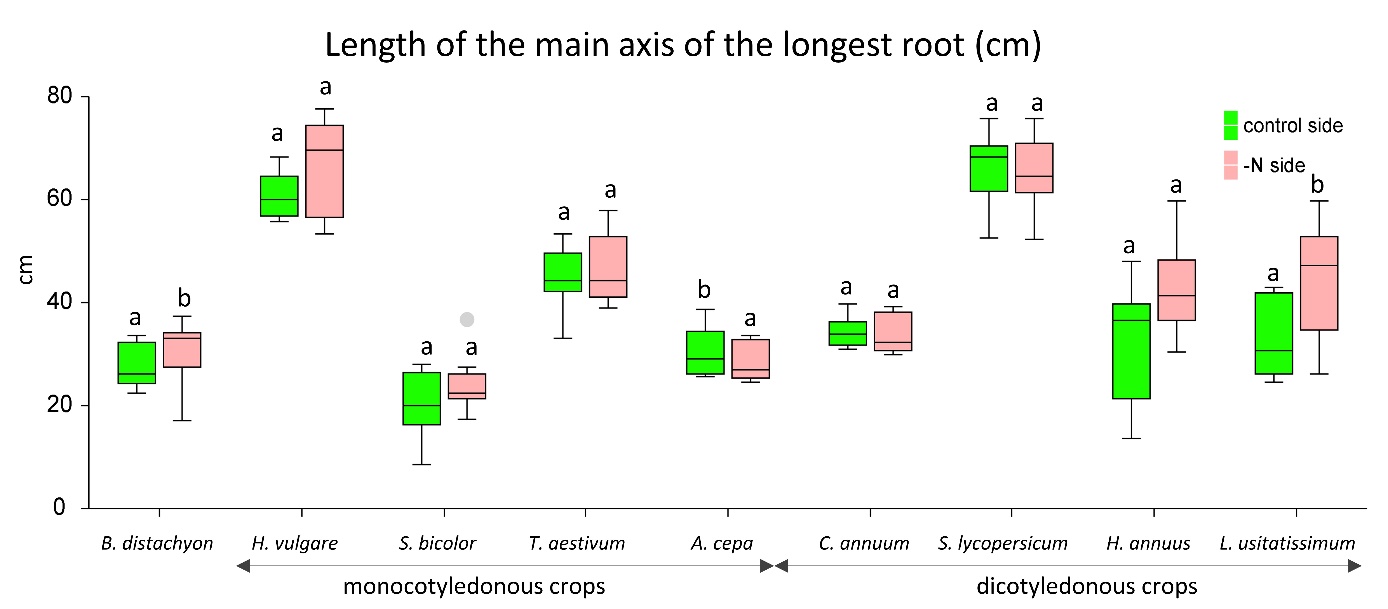


Supplementary Figure 2. Lengths of the main axis of the longest roots in the control and N-deficient part of the split-root system (mean ± SE). Wilcoxon Signed-Rank test, different letters indicate significant differences between the halves of the divided root system of a given species, p<0.05 (*n*=5-12).


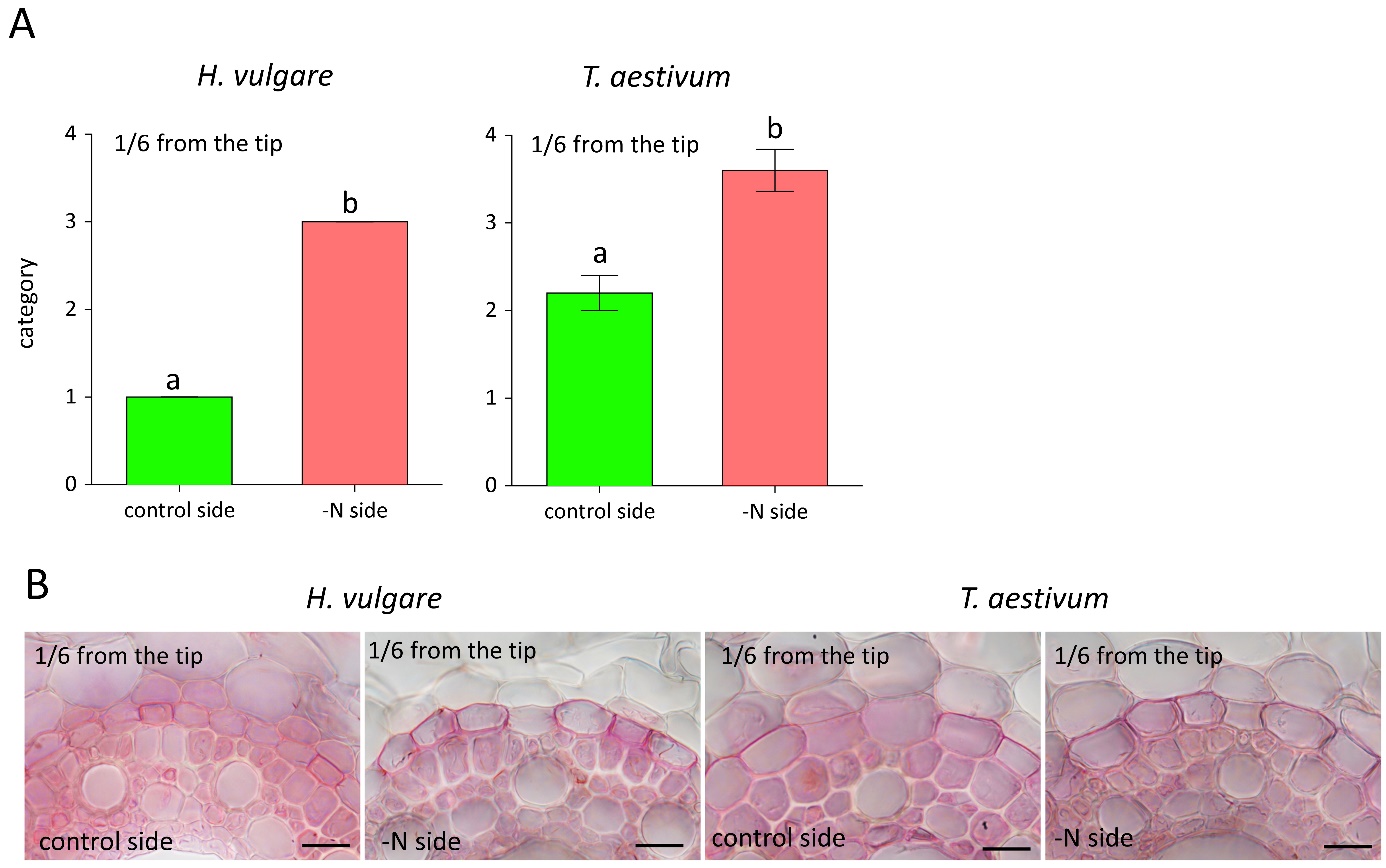


Supplementary Figure 3. Local root responses of endodermal suberisation to N deficiency in *H. vulgare* and *T. aestivum* in split-root hydroponics. **(A)** The incidence of cells with SL in the endodermal layer (mean ± SE). The category 0-4: 0 (complete absence), 1 (up to ⅓ of cells), 2 (⅓ to ⅔ of cells), 3 (⅔ of cells up to almost complete), and 4 (complete presence). Different letters indicate significant differences (Wilcoxon Signed-Rank test p<0.05, *n*=5). **(B)** Endodermal SL in control and N-deficient side of split root systems. Sudan Red7B, brightfield. Scale bars 20 µm. The relative positions along the root axis where roots were sectioned and analysed are indicated in the graphs and photographs. Suberin lamellae stained with Sudan Red 7B are visible as red-colored cell wall layer in endodermis.


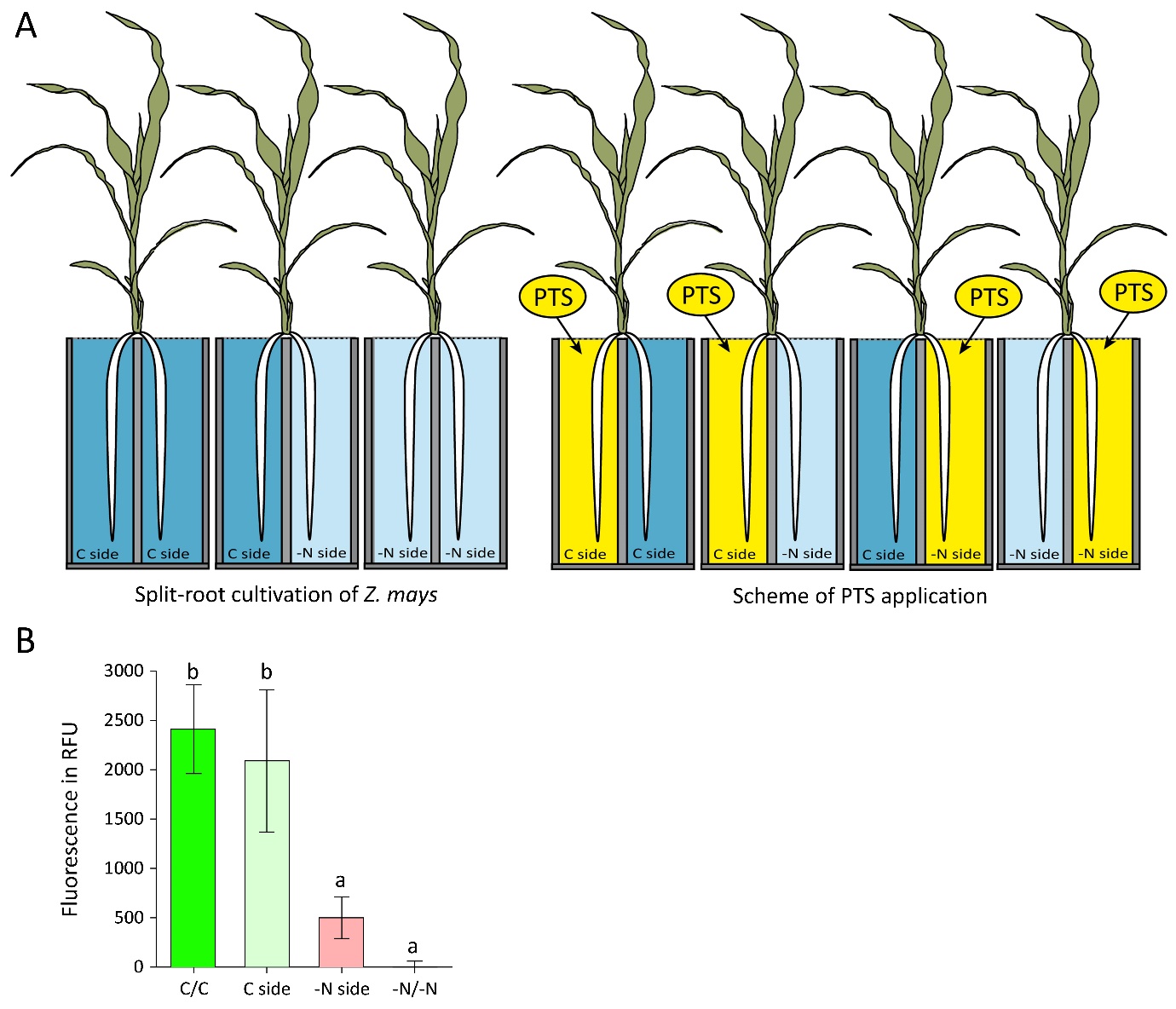


Supplementary Figure 4. Root permeability of *Z. mays* under locally applied N deficiency tested with PTS apoplastic tracer. **(A)** Scheme of the 14-day cultivation and PTS application. **(B)** PTS fluorescence in shoots after 24h incubation in PTS solution (mean ± SE). Data are expressed as the difference from the respective negative controls (plant without PTS treatment). Different letters indicate significant differences (Kruskal-Wallis Z multiple comparison test, p<0.05, *n*=6-8).
